# Case Report: Surgical Management of Painful Manubriosternal Pseudoarthrosis

**DOI:** 10.3389/fsurg.2021.640089

**Published:** 2021-03-09

**Authors:** Ronit Bar-Haim, Haim Shtarker, Seema Biswas, Igor Waksman, Edward Altman

**Affiliations:** ^1^Department of Surgery B, Galilee Medical Center, Nahariya, Israel; ^2^Department of Orthopedics, Galilee Medical Center, Nahariya, Israel; ^3^Faculty of Medicine in Tzfat, Bar Ilan University, Ramat Gan, Israel

**Keywords:** manubriosternal joint, pseudoarthrosis, chronic pain, surgery, weightlifter

## Abstract

A 31-year-old male amateur bodybuilder presented with a 2-year history of chronic pain over the sternum and a clicking sensation in the chest wall on movement. Ultrasound, computed tomography (CT), and magnetic resonance imaging (MRI) showed no cause for his symptoms. Dynamic ultrasound scan performed at a specialist sports center revealed pseudoarthrosis of the manubriosternal joint (MSJ). After a period of conservative management (rest and analgesia), he failed to improve and underwent debridement and fusion of the MSJ with plates and screws. At follow-up 23 months later, he remains pain-free and has returned to weight lifting and bodybuilding.

## Introduction

Motor vehicle crashes and chest seat belt restraints are the most common causes of sternal fracture. Reports of sternal injury among sportsmen and athletes are less common, however, with fracture resulting from non-contact trauma described in only sporadic cases in the literature (1–5).

While surgery for sternal fracture after acute trauma is rare, surgical repair of painful chronic non-union after sternal fracture has been described (6), as has surgery for chest pain after poorly healed median sternotomy in cardiac surgery resulting in malunion or pseudoarthrosis (7).

We describe the management of painful manubriosternal joint (MSJ) disruption in an amateur weight lifter and describe the challenge of diagnosis, pitfalls in imaging, and a technique of repair to render the patient symptom-free and able to resume his hobby.

## Technique

**Figure 1:**
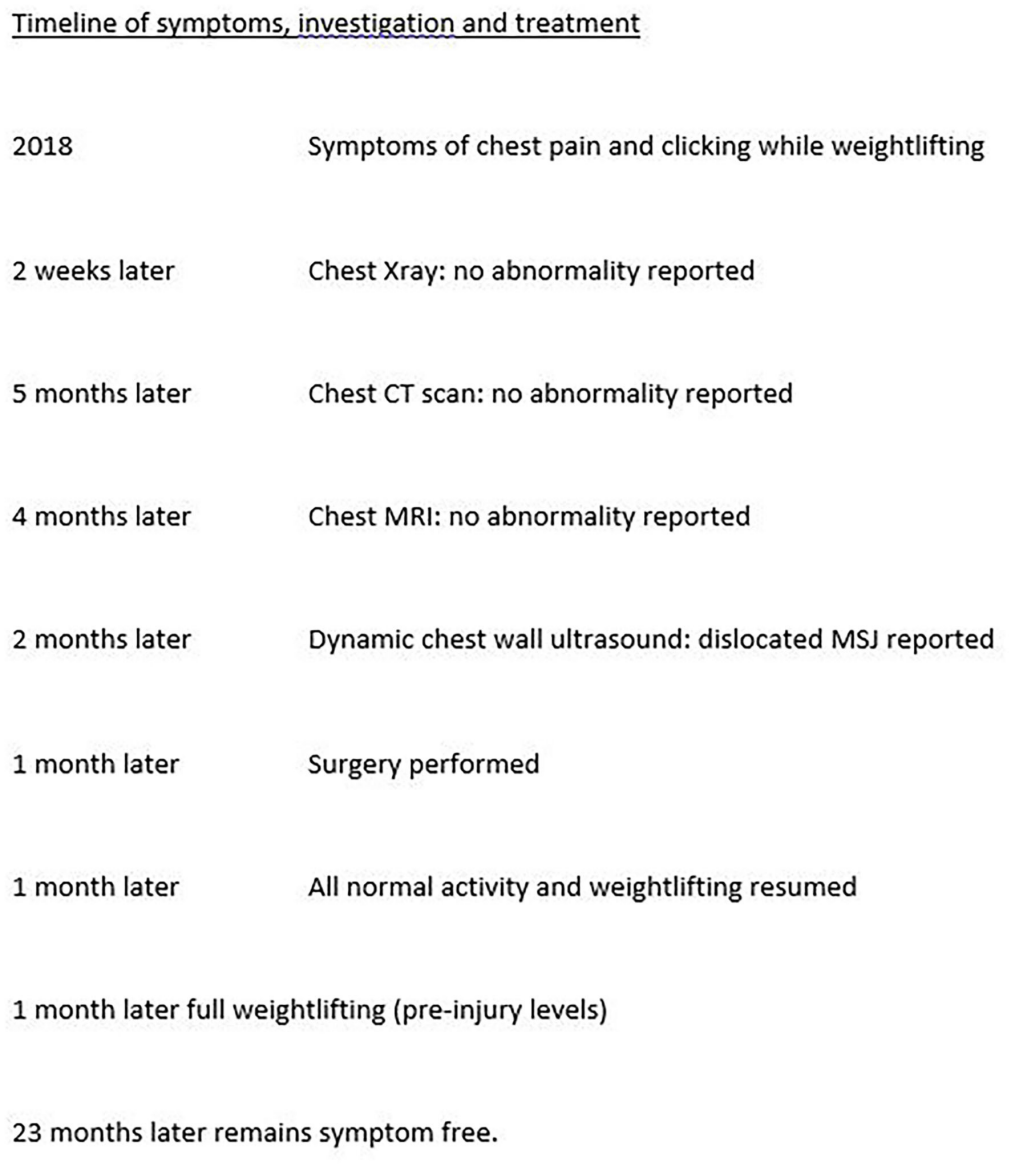
Timeline of symptoms, investigation, and treatment.

A 31-year-old man, a bodybuilder, presented with a 2-year history of chronic sternal pain and clicking on movement. On examination, he appeared pain-free and had an athletic body with very well-developed muscles of the torso and upper limbs. On the chest wall, a non-tender, bony thickening was palpable at the MSJ. After repeat consultation and a normal thoracic ultrasound scan, computed tomography (CT) and magnetic resonance imaging (MRI) scans were reported to show no abnormality. The patient's symptoms remained, and after 2-years of repeated consultations (see timeline in Figure 1), a dynamic ultrasound scan performed in a specialist sports medical center showed, in the first static phase, in the upright sitting position, a diastasis of 7.5 mm with deformation and elongation of the cartilage of 1.6 cm at the MSJ. The second phase, performed with the patient sitting forward with both arms raised, demonstrated unstable articulation of the manubrium on the body of the sternum with fibrosis of the MSJ—a pseudoarthrosis. Review of the previous MRI scan confirmed the pseudoarthrosis with a visible line of synovial fluid between the manubrium and the body of the sternum. These findings were consistent with chronic inflammation resulting from weight lifting. After a period of conservative treatment with rest and non-steroidal anti-inflammatory drugs, as there was no improvement, surgery was performed.

Under general anesthesia, a longitudinal midline sternal incision was made. Pathological movement of the MSJ was noted, with proliferation of synovial and fibrotic tissue. All abnormal fibrocartilaginous and synovial tissue was debrided using a high-speed burr. The MSJ was fixated with two 2-inch longitudinal titanium plates, parallel to each other, and three pairs of locked (2.7 cm) and non-locked (2.7 cm) screws to increase stability while permitting initial slight movement with breathing. Autologous bone graft was performed by harvesting cancellous bone from the iliac crest and inserting this into the defect at the MSJ. At the end of the procedure, a stability check was performed with no pathological movement of the MSJ. The patient made an uneventful postoperative recovery and was discharged 7 days later after completion of a course of physical therapy. He was pain-free and without any limitation of movement. He returned to normal activities 2 weeks later. After 1 month, he was regularly lifting weights at preinjury levels. At 23 month follow-up, the patient remains pain-free and has returned to regular exercise and weight lifting.

## Comment

While much has been written about the management of sternal fractures after blunt trauma and non-union after median sternotomy for cardiac surgery, guidance on the management of MSJ pseudoarthrosis remains relatively scarce.

The first challenge is in understanding the pathology in the absence of a single traumatic incident:

There are two known mechanisms of MSJ dislocation or disruption; both entail the high-energy trauma expected in a motor vehicle crash. The first type of injury results from direct impact with posterior displacement of the body of the sternum. The second and more common injury type follows flexion–compression trauma of the thorax and neck where the neck is forcefully hyperflexed while the chin, clavicles, or ribs displace the manubrium posteriorly.

In the absence of a single incidence of major trauma, a careful history of how and when the pain started is important: the mechanism here is repeated low-level trauma or stress on the chest wall. A key feature of the symptoms is clicking of the fibrocartilaginous pseudoarthrosis on movement of the unstable MSJ [also reported by (4), in a rugby player complaining of chest pain over 2-years].

**Figure 2:**
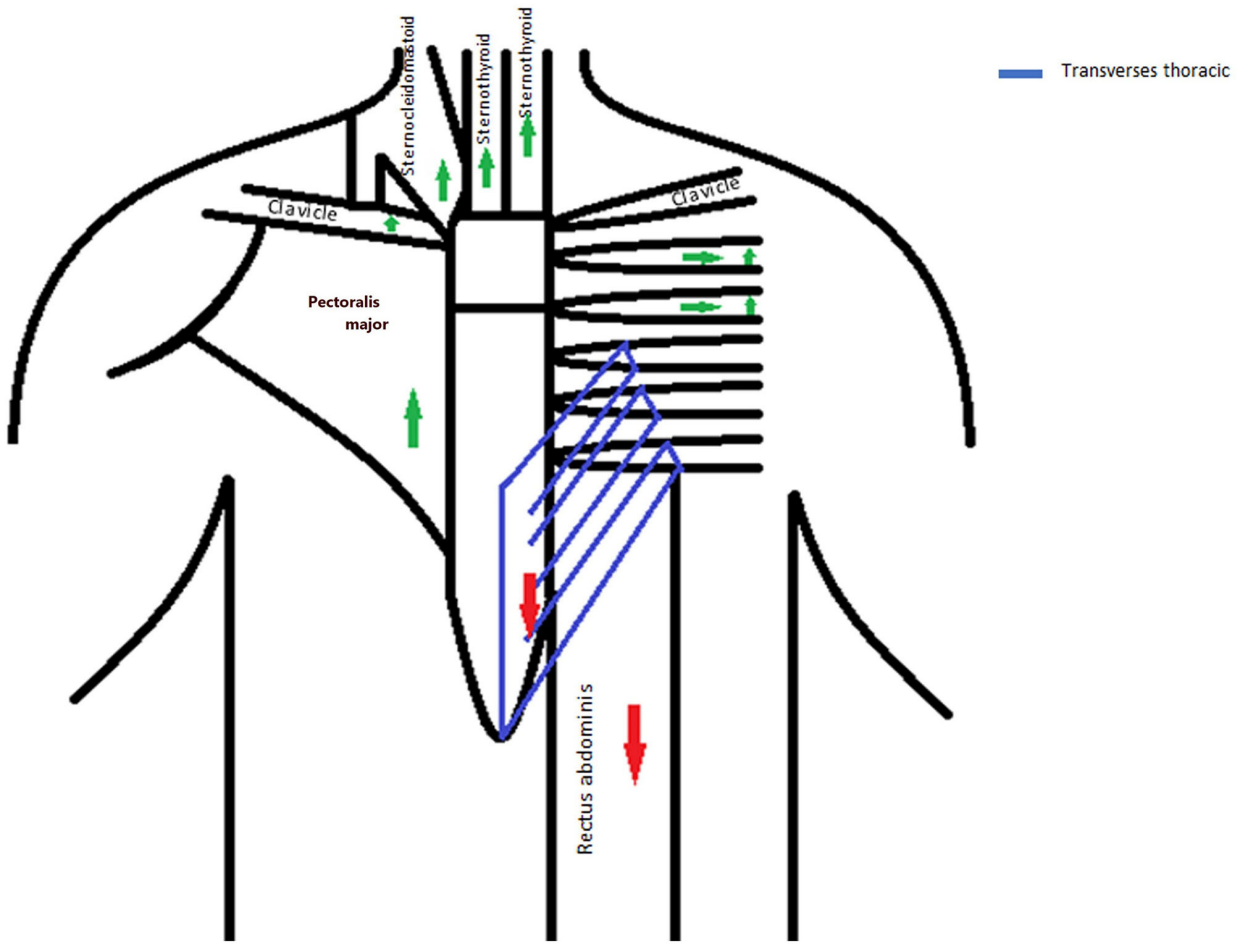
Anatomical forces acting on the manubriosternal joint.

The strength and stability of the MSJ rely on the attachment of the clavicles and first rib to the body of the manubrium and the strong ligamentous attachments of the second ribs to the MSJ. These attachments result in a non-mobile joint. In our patient, years of bodybuilding and weight lifting resulted in these muscles and ligaments stretched repeatedly under considerable force in opposite directions. Cranial stretching involved the attachment of the manubrium to the spine through the first and second ribs and the attachment to the shoulders through the clavicles, the sternal head of the sternocleidomastoid and part of the pectoralis major attached anteriorly to the manubrium and sternum, and the sternohyoid and sternothyroid muscles attached posteriorly to the manubrium. Caudally, stretching in the opposite direction involved the transverse thoracis muscle attached to the posterior surface of the body of the sternum and the strong rectus abdominis attached to the distal part of the sternum (Figure 2). Repetitive injury resulted in pseudoarthrosis of the MSJ.

Bilateral first rib fractures and pseudoarthrosis and callus formation at the thoracic inlet were described by Satija et al. (5) in a 17-year-old boy who complained of pain after 18 months of regular weight lifting. Zabaleta et al. (4) describe sternal pseudoarthrosis in a rugby player who similarly underwent debridement and titanium osteosynthesis.

**Figure 3:**
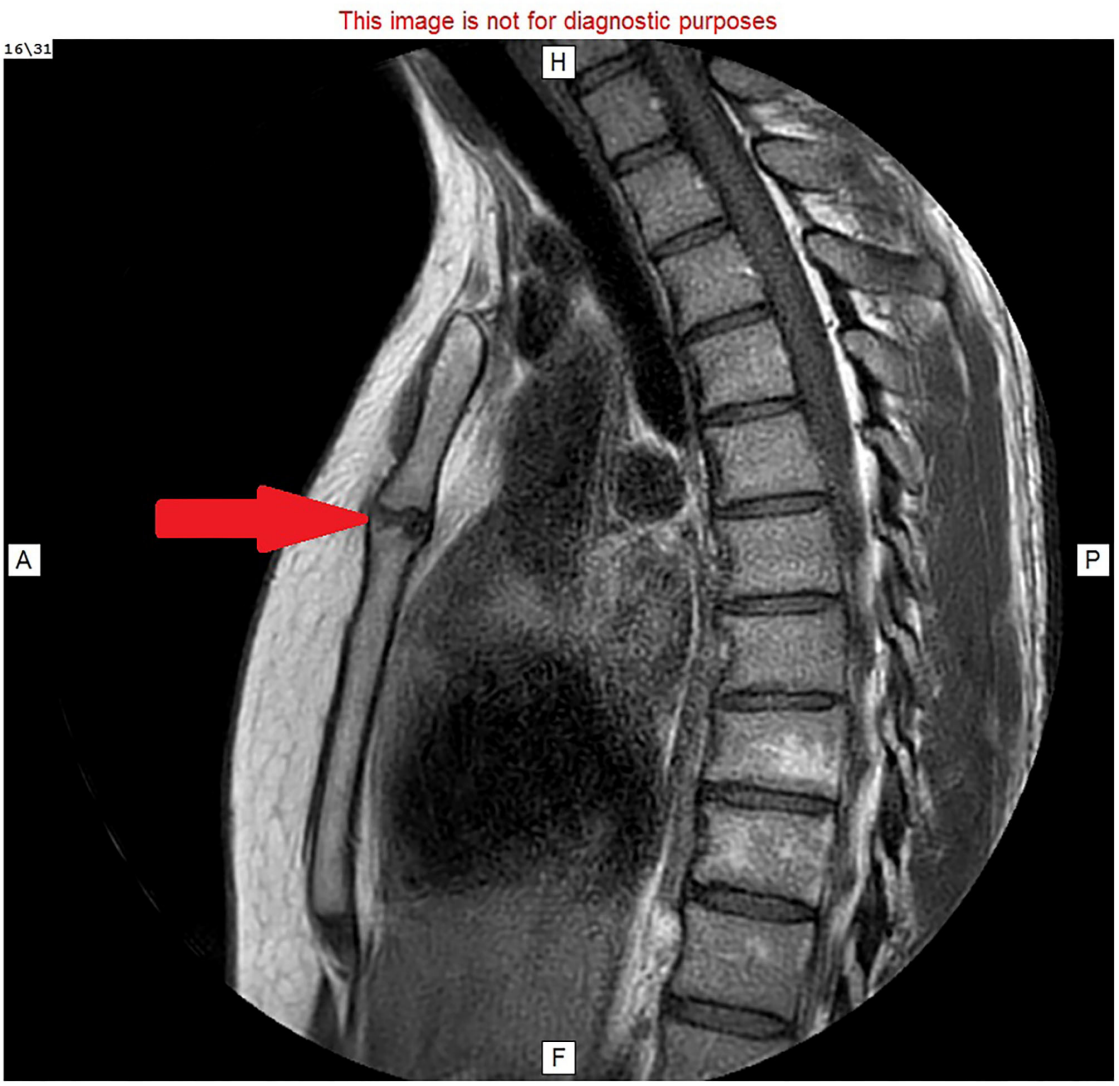
MRI image: T1 with faset and gadolinium contrast, sagittal view, showing hyperemic fluid in the manubriosternal joint (red arrow).

**Figure 4:**
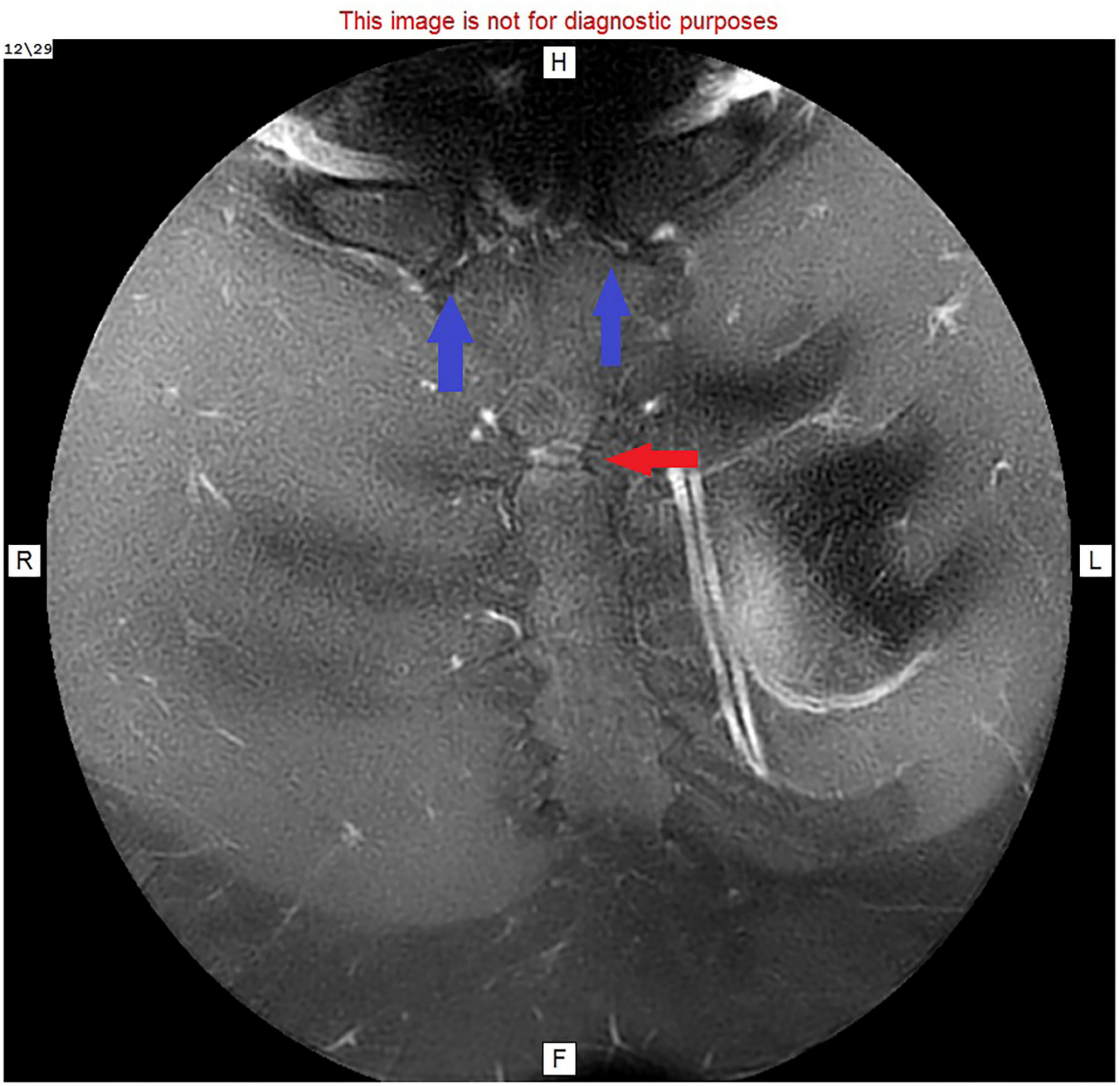
MRI image: T1 with faset and gadolinium contrast, cranial view, showing enhanced manubriosternal joint (red arrow) and normal sternoclavicular joints (blue arrows).

Rarer still are reported non-traumatic inflammatory lesions of the manubriosternal synchondrosis reported among athletes complaining of chronic post-exercise pain at the MSJ (2). While clinical practice in high-energy chest trauma is focused on thorough investigation to detect internal chest complications leading to cardiac, pulmonary, or vascular compromise, our emphasis is on the difficulty of diagnosis of chest wall complications resulting from relatively low-energy trauma. Dynamic testing was crucial in reaching the diagnosis. Thus, the second challenge was reaching the correct diagnosis through appropriate imaging:

Imaging findings of MSJ dislocation have been described (3), but pseudoarthrosis of the MSJ is rarely described in the literature. Interpretation of MRI scans without careful attention to the patient's history presents a challenge (Figures 3, 4). The key to diagnosis is functional or dynamic imaging, recreating the patient's symptoms while imaging the abnormal movement or articulation of the MSJ.

The final challenge was in selecting an appropriate operative technique to render the patient pain-free with a fused stable MSJ able to withstand the resumption of sporting activity:

While most sternal injuries are managed conservatively, there are three key indications for surgery: (I) the presence of a sternal deformity, (II) loss of sternal continuity for a period of more than 6 weeks, and (III) persistence of chest pain for 2–8 weeks after trauma.

Surgical fixation of the dislocated MSJ using plates and screws has yielded demonstrably better results than that achieved with wire fixation (8). The superficial positioning of the sternum beneath the chest wall skin limits the thickness of plates that can be used; thus, thin plates are used. Parallel positioning of the plates, with both locked and non-locked screw fixation, further increases stability while permitting initial movement with breathing. We recommend the use of two plates for two reasons: to limit movement (to prevent rotational motion between the manubrium and sternum) and to maintain the strength of the fixation. The use of locked screws makes for a more rigid fixation, especially in thin bone—an important consideration in treating pseudoarthrosis. To date, we have not experienced complications, including infection, using chest wall plate and screw fixation after primary surgery, albeit a relatively rare procedure in our department. Seroma and infection have been reported after rib fixation, with infection rates in small series as high as 7% (9). Infection is the key disadvantage of plate and screw fixation, but the stable fixation this gives surpasses pin or wire fixation—also potentially complicated by infection.

Twenty-three months after surgery, the patient reports no further clicking or pain, and, on examination, there is no further evidence of movement at the MSJ.

## Data Availability Statement

The datasets presented in this article are not readily available because they are stored in confidential patient medical records. Requests to access the datasets should be directed to ronitbarh@gmail.com.

## Ethics Statement

Written informed consent was obtained from the individual(s) for the publication of any potentially identifiable images or data included in this article.

## Author Contributions

All authors listed have made a substantial, direct and intellectual contribution to the work, and approved it for publication.

## Conflict of Interest

The authors declare that the research was conducted in the absence of any commercial or financial relationships that could be construed as a potential conflict of interest.
